# Association of Hepatitis B and C Virus with the Risk of Coronary Artery Disease and Cerebrovascular Disease in Patients with Hepatocellular Carcinoma

**DOI:** 10.3390/jcm12072602

**Published:** 2023-03-30

**Authors:** Meng-Chuan Lu, Ying-Hsuen Wu, Chi-Hsiang Chung, Hsuan-Hwai Lin, Tsai-Yuan Hsieh, Peng-Jen Chen, Wu-Chien Chien, Hsuan-Wei Chen

**Affiliations:** 1 Division of Gastroenterology, Department of Internal Medicine, Tri-Service General Hospital, National Defense Medical Center, 325, Sec 2, Cheng-Kung Road, Taipei Neihu 114, Taiwan; 2 Department of Ophthalmology, China Medical University Hospital, School of Medicine, College of Medicine, China Medical University, Taichung City 404, Taiwan; 3 Department of Medical Research, Tri-Service General Hospital, Taipei 114, Taiwan; 4 School of Public Health, National Defense Medical Center, Taipei 114, Taiwan

**Keywords:** hepatocellular carcinoma, hepatitis B, hepatitis C, coronary artery disease, cerebrovascular disease

## Abstract

Background: Hepatocellular carcinoma accounts for approximately 90% of primary liver cancers and hepatitis virus was believed to have the potential for altering the pathogenesis of arteriosclerosis. However, the influence of the hepatitis virus on coronary artery disease or cerebral vascular disease remains unclear. This study used the Taiwan National Health Insurance Research Database to clarify the virus-associated risk of coronary artery disease and cerebral vascular disease in patients with hepatocellular carcinoma (HCC). Methods: A total of 188,039 HCC individuals, age 20 years or older, were enrolled from the Longitudinal Health Insurance Database between 2000 and 2017 for cohort analysis. A total of 109,348 with hepatitis B virus (HBV) infection, 37,506 with hepatitis C virus (HCV) infection, 34,110 without HBV or HCV, and 7075 with both HBV and HCV were recorded. Statistically, propensity score matched by sex, age, and index year at a ratio of 15:5:5:1 and a sensitivity test using multivariable Cox regression were used. Results: The risk of coronary artery disease in the HCV-related HCC group was 1.516-fold (95% CI: 1.328–2.034, *p* < 0.001) higher than in the HBV-related HCC group, followed by the HBV/HCV-related HCC group and the non-B/C HCC group; the cerebral vascular disease risk in the HCV-related HCC group was 1.467-fold higher than in the HBV-related HCC group (95% CI: 1.335 to 1.786, *p* < 0.001), followed by the HBV/HCV-related HCC group and the non-B/C HCC group. Conclusion: Hepatitis C virus infection was found to have a higher risk of developing coronary artery disease or cerebral vascular disease in patients with hepatocellular carcinoma. For patients with hepatocellular carcinoma, our findings warrant the importance in preventing artherosclerotic disease in the setting of hepatitis C virus infection.

## 1. Introduction

Hepatocellular carcinoma (HCC) accounts for approximately 90% of primary liver cancers, making it the third leading cause of cancer-related deaths worldwide. HCC primarily occurs in patients with underlying liver disease, mainly due to viral infection or alcohol misuse [1]. Coronary artery disease (CAD) and cerebrovascular disease (CVD) are also major public health issues because of their considerable impact on disability and mortality in adults worldwide [2,3]. Given that nonalcoholic fatty liver disease may be related to atherosclerosis [4] and that the relationship between hepatitis virus and atherogenesis remains unclear, some types of hepatitis virus may influence the risk of CAD or CVD in patients with HCC. Some studies have indicated that no significant relationship exists between hepatitis B (HBV) infection and coronary atherosclerosis [5,6], whereas others have indicated that HBV infection is associated with lower risks of CAD [7] and stroke [8,9]. Furthermore, many systematic reviews and meta-analyses have indicated that hepatitis C virus (HCV) infection is a risk factor for CAD [10,11,12] and CVD [12,13,14,15,16,17]. The potential pathogenesis of reducing atherogenic risk by HBV infection includes that which disturbs lipid metabolism and reduces systemic inflammation [6,18,19,20,21]. On the contrary, HCV infection increases the atherogenesis by initiating chronic inflammation [22,23]. Several studies have shown that consumption of moderate alcohol (<30 g/day) may be helpful in preventing coronary artery disease [24]. However, high dose alcohol consumption also induces oxidative stress and a wide variety of inflammatory markers [25]. Together, these findings indicate that the hepatitis virus may alter the pathogenesis of arteriosclerosis.

With advancements in the development of the hepatitis B vaccine and direct-acting antiviral agents, the incidence of HCC in patients without HBV or HCV infection (NBNC HCC) is gradually increasing [26,27]. To further our understanding of the influence of the hepatitis virus on CAD and CVD risk in patients with HCC, CAD/CVD risk in patients with NBNC HCC should also be examined. In this study, we used a nationwide database to analyze the risk of CAD and CVD in patients with viral hepatitis-related HCC and NBNC HCC.

## 2. Methods

### 2.1. Data

Taiwan’s National Health Insurance Research Database (NHIRD) was established in 1995, and it covers the Health Insurance Administration claims data, including inpatient, outpatient, and emergency data, of >99% of the residents in Taiwan. In the present study, data from the NHIRD were used. The investigation protocols were approved by the official peer review committee of Tri-Service General Hospital. The diagnoses were made according to the International Classification of Diseases, Ninth Revision, Clinical Modification (ICD-9-CM) [28] and the International Classification of Diseases, Tenth Revision, Clinical Modification (ICD-10-CM) [29]. The National Health Insurance Administration approved the use of the Longitudinal Health Insurance Database in this study.

### 2.2. Study Cohort

For this retrospective cohort study, we obtained outpatient and inpatient data of patients with HCC with or without HBV or HCV infection from the NHIRD between 1 January 2000 and 31 December 2017. HCC was identified using ICD-9-CM code 155.0 and ICD-10-CM codes C22.0–C22.4. HBV was identified using ICD-9-CM codes 070.20–070.23 and 070.30–070.33; ICD-10-CM code B16; a prescription of an anti-HBV agent, including lamivudine, adefovir, entecavir, telbivudine, or tenofovir disoproxil fumarate; or positive results for HBV viral load DNA quantitative amplification test. HCV was identified using ICD-9-CM codes 070.41, 070.44, 070.51, and 070.54; ICD-10-CM code B18.2; or positive results for HCV viral load RNA quantitative amplification test or HCV genotyping test (polymerase chain reaction). Patients with HCC who did not have any HBV/HCV-associated ICD codes or anatomical therapeutic chemical codes were included in the NBNC HCC group.

**Figure 1:**
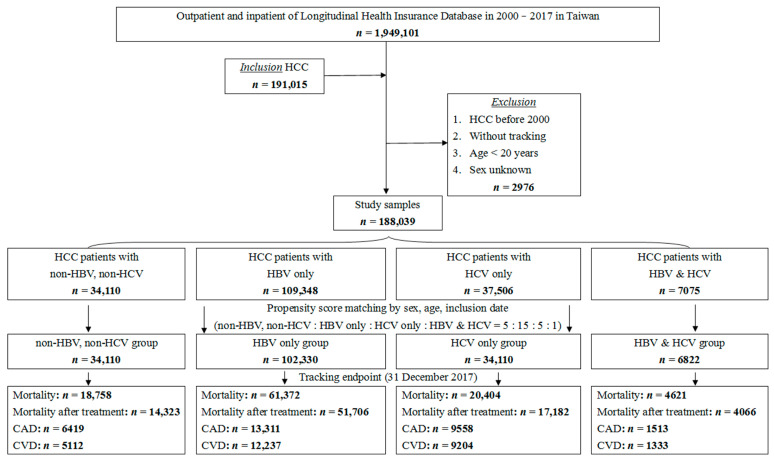
Flowchart of the study. The abbreviations: hepatocellular carcinoma (HCC); hepatitis B (HBV); hepatitis C (HCV); coronary artery disease (CAD); cerebrovascular disease (CVD).

Initially, the data of 191,015 patients were collected; of them, 2976 individuals were excluded because their conditions were diagnosed before 1 January 2000, or <20 years of age. Furthermore, cases of unknown sex were also excluded (Figure 1). Finally, our study included data from 188,039 patients: 109,348 with HBV-related HCC, 34,110 with NBNC HCC, 37,506 with HCV-related HCC, and 7075 with both HBV and HCV (HBV/HCV-related HCC). Subsequently, we used propensity score matching by sex, age, and index year at a ratio of 15:5:5:1, pertaining to 102,330, 34,110, 34,110, and 6822 individuals, respectively. Because of the lower risk of atherogenic disease in patients with HBV disclosed by some latest studies [7,8,9] and NBNC HCC patients shared more similar risk factors of CAD/CVD such as diabetes mellitus, hypertension, dyslipidemia, and lifetime alcohol consumption [26,30], we defined the group of patients with HBV-related HCC as the control group and the other three as the comparison groups.

The study endpoint was a diagnosis of CAD (ICD-9-CM codes: 410, 411, 412, 413, and 414) and CVD (ICD-9-CM codes: 430, 431, 432, 433, 434, 435, 436, 437, and 438) during the follow-up period. Among all the enrolled patients, if there were CAD or CVD events, it was the endpoint date for those patient patients; for patients without either CAD or CVD events, the endpoint date was the time of death or the day of 31 December 2017, the last follow-up time of this study. Relevant covariates included sex, age, insured premium, and baseline comorbidities diagnosed before the index entry date, such as dyslipidemia (ICD-9-CM code: 272.xx), diabetes (ICD-9-CM code: 250), hypertension (ICD-9-CM codes: 401.9 and 405.99), chronic obstructive pulmonary disease (ICD-9-CM codes: 491.20, 493.20, 496), cirrhosis (ICD-9-CM code: 571), and chronic kidney disease (ICD-9-CM code: 585.9).

### 2.3. Statistical Analysis

All statistical analyses were conducted using SPSS version 21.0 (IBM, Armonk, NY, USA). A chi-squared test was used to compare categorical variables. Univariable and multivariable Cox proportional hazard models were used to determine the risk of CAD and CVD, and subgroup analyses were performed to compare differences in the risk between the HBV-related, HCV-related, and NBNC HCC groups. The results are presented as adjusted hazard ratios (aHRs) with 95% confidence intervals (CIs). Stratified analyses were conducted to compare the effects of hepatitis virus infection, sex, age, region, socioeconomic status, and cost on HCC development. Between-group differences in the cumulative incidence risk of CAD or CVD were illustrated using Kaplan–Meier plots with a log-rank test. Statistical significance was set at a two-tailed *p* value of <0.05.

## 3. Results

The clinical baseline characteristics of the included patients are summarized in Table 1. We included 102,330 (57.69%) patients in the HBV-related HCC group (the study group), and 34,110 (19.23%), 34,110 (19.23%), and 6822 (3.85%) patients in the NBNC, HCV-related, and HBV/HCV-related HCC groups (the comparison groups), respectively.

The mean age of the NBNC, HCV-related, and HBV/HCV-related HCC groups was 55.21 ± 19.84, 55.24 ± 19.99, and 55.29 ± 20.03 years, respectively, whereas that of the matched HBV-related HCC group was 55.24 ± 19.96 years (*p* = 0.999). The most prevalent comorbidities, including dyslipidemia, diabetes, hypertension, chronic obstructive pulmonary disease, cancer, and cirrhosis, were more common in the HBV/HCV-related HCC cohort than in other cohorts (all *p* < 0.0001, Table 1).

After adjustment for sex, age, insured premium, dyslipidemia, diabetes, hypertension, chronic obstructive pulmonary disease, cancer, cirrhosis, urbanization level, and level of care with the Cox proportional hazards regression model, the risk of CAD in the HCV-related HCC group was 1.516-fold (95% CI: 1.328–2.034, *p* < 0.001) higher than in the HBV-related HCC group, followed by 1.497-fold (95% CI: 1.292–1.995, *p* < 0.001) in the HBV/HCV-related HCC group and 1.153-fold (95% CI: 0.917–1.773, *p* = 0.094) in the NBNC HCC group (Table 2).

Compared with the HBV-related HCC group, the aHR of CVD was the highest in the HCV-related HCC group (aHR = 1.467, 95% CI: 1.335 to 1.786, *p* < 0.001), followed by the HBV/HCV-related HCC group (aHR = 1.424, 95% CI: 1.290–1.721, *p* < 0.001) and the NBNC HCC group (aHR = 1.083, 95% CI: 0.887–1.527, *p* = 0.189).

Dyslipidemia, diabetes, hypertension, chronic kidney disease, cirrhosis, and older age (50–90 years old) were identified as independent prognostic factors in both CAD and CVD prevalence. Moreover, the higher urbanization level and hospital center level of care were related to higher aHR of both CAD and CVD (Table 2).

**Figure 2:**
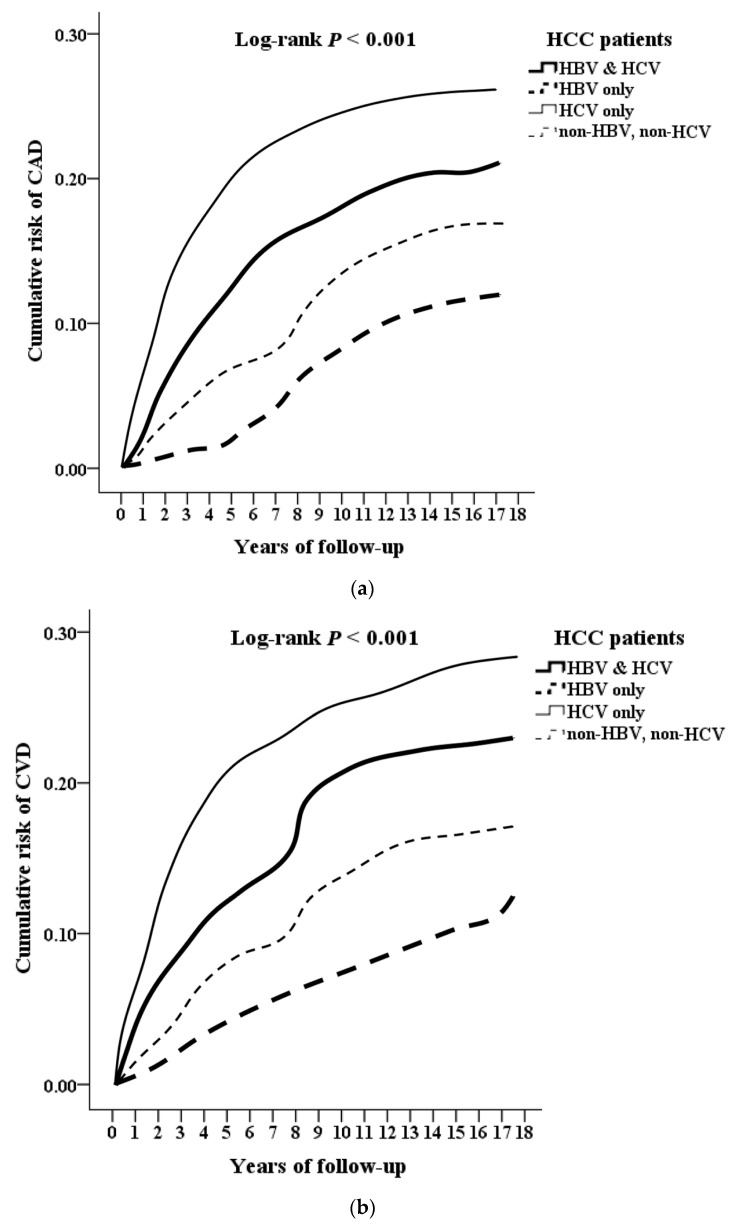
(**a**). Kaplan–Meier for cumulative risk of CAD among HCC patients, aged 20, and over stratified by HBV and HCV with log-rank test. The abbreviations: hepatocellular carcinoma (HCC); hepatitis B (HBV); hepatitis C (HCV); coronary artery disease (CAD). (**b**). Kaplan–Meier for cumulative risk of CVD among HCC patients, aged 20. and over stratified by HBV and HCV with log-rank test. The abbreviations: hepatocellular carcinoma (HCC); hepatitis B (HBV); hepatitis C (HCV); cerebrovascular disease (CVD).

Kaplan–Meier analysis revealed that the HBV-related HCC group exhibited a significantly lower cumulative CAD and CVD incidence than the other groups (log-rank *p* < 0.001), with the HCV-related HCC group exhibiting the highest cumulative incidence (log-rank *p* < 0.001; Figure 2a,b).

One-way ANOVA and Scheffe post hoc test analysis revealed that the HBV-related HCC and HCV-related HCC groups had the longest and shortest mean duration to CAD (5.27 ± 5.31 and 4.44 ± 4.57 years, respectively; *p* = 0.018; Table 3). Similarly, the mean duration to CVD was 5.23 ± 5.28 and 4.41 ± 4.50 years, respectively (*p* = 0.007; Table 4).

## 4. Discussion

This is the first study to use a nationwide population database to evaluate the association of hepatitis B and C virus with the risk of CAD and CVD in patients with HCC. Patients with HBV-related HCC had the lowest risk of both CAD and CVD among all cohort studies, followed by patients with NBNC HCC, HBV/HCV-related HCC, and HCV-related HCC.

Atherosclerosis is a multifocal and immunoinflammatory disease affecting medium to large arteries caused by lipids; the most harmful consequences of atherosclerosis include CAD and CVD [31,32]. The lower risk of atherogenesis in patients with HBV-related HCC might be attributed to three factors: altered lipid metabolism, inflammation status, and immune system status. First, acute or chronic hepatitis due to HBV infection may disturb lipid metabolism and then reduce multiple atherogenic cardiometabolic risk factors, such as triglycerides, cholesterol, and lipoprotein [18,19,20,21]. Second, systemic inflammation facilitates endothelial dysfunction and arterial atherosclerosis. De-Yan Tong et al. reported that HBV infection was negatively correlated with systemic inflammation, as evaluated using C-reactive protein [6]. Third, HBV infection may induce some cytokines to protect the vascular endothelium, such as hepatocyte growth factor [33,34,35]. However, compared to our results, some studies did not find a significant association between risk of atherogenesis and HBV infection patients [5,6]. One previous study from a population of high HBV prevalence further expressed that HBV serological markers including hepatitis B surface antigen, hepatitis B surface antibody, hepatitis B e antigen, hepatitis B e antibody and hepatitis B core antibody were not significantly related with CAD [6].

In comparison, patients with HCV-related HCC had a significantly higher risk of CAD and CVD, which may be due to the following mechanisms. First, HCV structural and nonstructural proteins play major roles in initiating and maintaining chronic inflammation [22,23], which then promotes liver fibrogenesis [36]. A higher prevalence of atherosclerosis observed in chronic HCV infection has been correlated with liver fibrosis [37,38]. Second, chronic HCV infection is an independent predictor of cerebrovascular deaths, likely due to high serum HCV RNA levels [14]. Moreover, Shoeib et al. indicated that multivessel affection was found in 47.8% of HCV-positive patients compared with 22.2% of HCV-negative patients [39]. In a previous systematic review and meta-analysis conducted by Lee KK et al., considerable geographical variation was identified in the burden of cardiovascular disease attributable to HCV, with the highest burden observed in South Asia, Eastern Europe, North Africa, and the Middle East, which had geographical proximity to our study [12].

We supposed that patients with viral hepatitis-related HCC might have the original characteristics in the atherogenic mechanism. Our study confirmed that for patients with hepatitis virus B or C infection, the prevalence of CAD/CVD still had the same trend after HCC occurrence.

Our data also indicated that patients with NBNC HCC had a higher CAD/CVD risk than the HBV-related HCC group. This may have been because of alcohol use or metabolic syndrome; however, because these data are not available in the NHIRD, we could not perform a related analysis. A Korean study reported that heavy alcohol use was the most common potential etiology in NBNC HCC [30], and a Japanese nationwide survey of people with NBNC HCC found that in addition to older age, obesity, comorbidities of diabetes mellitus, hypertension, and dyslipidemia, lifetime alcohol consumption may be a crucial risk factor for this group [26]. Because of cultural similarities and geographical proximity, alcohol consumption and metabolic syndrome may also explain the higher risk of CAD and CVD in our cohort of Taiwanese patients with NBNC HCC.

Although patients with HBV/HCV coinfection tend to have more severe liver injury than patients with HBV or HCV monoinfection due to a higher probability of liver cirrhosis and hepatic decompensation [40], the potential protective effect of HBV infection on atherogenesis might also explain the lower prevalence of CAD/CVD in this group than that in the HCV-related HCC group in our study.

## 5. Limitations

The three primary strengths of our cohort study include the presentation of the incidence trends in the most recent decade between 2000 and 2017, control of comorbidities, and the use of national data with a large sample size. However, there are a number of limitations in the present study. First, the NHIRD lacks information on patients’ risk factors and other unmeasured confounders for atherogenic disease (e.g., dietary habit, alcohol consumption, tobacco smoking, body mass index). Second, the NHIRD does not provide data on associated laboratory data results, including liver function tests, serum albumin level, serum HBV or HCV viral load, and serum C-reactive protein levels, or imaging data, including abdominal computed tomography or magnetic resonance imaging data. Without these data, tumor stage, Child–Pugh score, and inflammation level could not be well characterized. Third, we could not investigate the likely protective factors for atherogenic disease, such as physical activity and prophylactic medicines prescribed for hypertension. Moreover, data on individual therapies involving anti-HBV or anti-HCV agents were unavailable, and the actual impact of viral infection might have been overestimated or underestimated. Finally, the retrospective cohort design precluded the analysis of cause–effect relationships. Further prospective randomized studies are warranted.

## 6. Conclusions

In this large population-based database study of patients with viral hepatitis-related HCC or NBNC HCC, we demonstrated that HBV-related HCC and HCV-related HCC were associated with significantly lower and higher risks of CAD/CVD, respectively. These findings have crucial clinical implications for reducing the associated burden in both CAD and CVD. For patients with HCC and HCV infection, our study reveals that more effort should be devoted to preventing CAD/CVD development.

## Figures and Tables

**Table 1:** Characteristics of study at the baseline.

HCC Patients	Overall		(1) Non-HBV, Non-HCV		(2) HBV Only		(3) HCV Only		(4) HBV and HCV		*p*
Variables	*n*	%	*n*	%	*n*	%	*n*	%	*n*	%	
Total	177,372		34,110		102,330		34,110		6822		
Sex											0.999
Male	137,124	77.31	26,370	77.31	79,110	77.31	26,370	77.31	5274	77.31	
Female	40,248	22.69	7740	22.69	23,220	22.69	7740	22.69	1548	22.69	
Age (years)	55.24 ± 19.96		55.21 ± 19.84		55.24 ± 19.96		55.24 ± 19.99		55.29 ± 20.03		
Age groups (years)											0.999
20–29	5174	2.92	995	2.92	2985	2.92	995	2.92	199	2.92	
30–39	17,316	9.76	3330	9.76	9990	9.76	3330	9.76	666	9.76	
40–49	44,954	25.34	8645	25.34	25,935	25.34	8645	25.34	1729	25.34	
50–59	49,868	28.11	9590	28.11	28,770	28.11	9590	28.11	1918	28.11	
60–69	25,506	14.38	4905	14.38	14,715	14.38	4905	14.38	981	14.38	
70–79	16,016	9.03	3080	9.03	9240	9.03	3080	9.03	616	9.03	
80–89	13,754	7.75	2645	7.75	7935	7.75	2645	7.75	529	7.75	
≥90	4784	2.70	920	2.70	2760	2.70	920	2.70	184	2.70	
Insured premium (NTD)											<0.001
<18,000	132,255	74.56	27,042	79.28	74,261	72.57	25,570	74.96	5382	78.89	
18,000–34,999	25,495	14.37	4365	12.80	14,771	14.43	5539	16.24	820	12.02	
≥35,000	19,622	11.06	2703	7.92	13,298	13.00	3001	8.80	620	9.09	
DM											<0.001
Without	145,387	81.97	28,137	82.49	85,077	83.14	27,055	79.32	5118	75.02	
With	31,985	18.03	5973	17.51	17,253	16.86	7055	20.68	1704	24.98	
HTN											<0.001
Without	142,064	80.09	27,444	80.46	82,718	80.83	26,823	78.64	5079	74.45	
With	35,308	19.91	6666	19.54	19,612	19.17	7287	21.36	1743	25.55	
COPD											<0.001
Without	168,442	94.97	32,347	94.83	97,779	95.55	31,951	93.67	6365	93.30	
With	8930	5.03	1763	5.17	4551	4.45	2159	6.33	457	6.70	
CKD											<0.001
Without	143,517	80.91	28,028	82.17	82,714	80.83	27,253	79.90	5522	80.94	
With	33,855	19.09	6082	17.83	19,616	19.17	6857	20.10	1300	19.06	
Dyslipidemia											<0.001
Without	173,325	97.72	33,261	97.51	100,046	97.77	33,390	97.89	6628	97.16	
With	4047	2.28	849	2.49	2284	2.23	720	2.11	194	2.84	
Cancer											<0.001
Without	150,223	84.69	29,647	86.92	86,129	84.17	28,871	84.64	5576	81.74	
With	27,149	15.31	4463	13.08	16,201	15.83	5239	15.36	1246	18.26	
Cirrhosis											<0.001
Without	144,323	81.37	28,313	83.00	83,540	81.64	27,086	79.41	5384	78.92	
With	33,049	18.63	5797	17.00	18,790	18.36	7024	20.59	1438	21.08	
Urbanization level											<0.001
1 (the highest)	54,979	31.00	9198	26.97	32,494	31.75	10,945	32.09	2342	34.33	
2	63,455	35.78	11,422	33.49	37,201	36.35	12,235	35.87	2597	38.07	
3	27,100	15.28	6057	17.76	15,091	14.75	5072	14.87	880	12.90	
4 (the lowest)	31,838	17.95	7433	21.79	17,544	17.14	5858	17.17	1003	14.70	
Level of care											<0.001
Hospital center	74,704	42.12	11,152	32.69	44,519	43.51	15,238	44.67	3795	55.63	
Regional hospital	59,270	33.42	12,428	36.44	34,724	33.93	10,438	30.60	1680	24.63	
Local hospital	43,398	24.47	10,530	30.87	23,087	22.56	8434	24.73	1347	19.74	

*p*: chi-square/Fisher exact test on category variables and t-test on continue variables. The abbreviations: hepatocellular carcinoma (HCC); hepatitis B (HBV); hepatitis C (HCV); coronary artery disease (CAD); cerebrovascular disease (CVD); New Taiwan dollar (NTD); diabetes mellitus (DM); hypertension (HTN); chronic obstructive pulmonary disease (COPD); chronic kidney disease (CKD).

**Table 2:** Factors of CAD/CVD by using multivariable Cox regression.

Outcomes	CAD				CVD			
Variables	aHR	95% CI	95% CI	*p*	aHR	95% CI	95% CI	*p*
HCC patients								
non-HBV, non-HCV	1.153	0.917	1.773	0.094	1.083	0.887	1.527	0.189
HBV only	Reference				Reference			
HCV only	1.516	1.328	2.034	<0.001	1.467	1.335	1.786	<0.001
HBV and HCV	1.497	1.292	1.995	<0.001	1.424	1.290	1.721	<0.001
Sex								
Male	1.151	1.075	1.199	<0.001	1.127	1.071	1.184	<0.001
Female	Reference				Reference			
Age groups (years)								
20–29	Reference				Reference			
30–39	1.011	0.908	1.099	0.515	1.041	0.956	1.121	0.440
40–49	1.064	0.971	1.135	0.370	1.083	1.017	1.167	0.037
50–59	1.115	1.047	1.158	0.002	1.159	1.099	1.232	<0.001
60–69	1.158	1.091	1.184	<0.001	1.177	1.120	1.233	<0.001
70–79	1.131	1.066	1.168	<0.001	1.148	1.089	1.222	<0.001
80–89	1.208	1.138	1.252	<0.001	1.196	1.124	1.247	<0.001
≥90	1.045	0.952	1.120	0.431	1.042	0.960	1.146	0.415
Insured premium (NTD)								
<18,000	Reference				Reference			
18,000–34,999	1.041	0.953	1.160	0.311	1.022	0.951	1.157	0.428
≥35,000	0.971	0.884	1.116	0.483	0.968	0.874	1.114	0.463
DM								
Without	Reference				Reference			
With	1.245	1.121	1.369	<0.001	1.257	1.166	1.353	<0.001
HTN								
Without	Reference				Reference			
With	1.368	1.226	1.483	<0.001	1.355	1.190	1.463	<0.001
COPD								
Without	Reference				Reference			
With	1.158	1.002	1.247	0.049	1.138	0.990	1.232	0.268
CKD								
Without	Reference				Reference			
With	1.168	1.097	1.257	<0.001	1.178	1.098	1.265	<0.001
Dyslipidemia								
Without	Reference				Reference			
With	1.096	0.910	1.214	0.430	1.071	0.887	1.201	0.521
Cancer								
Without	Reference				Reference			
With	1.327	1.141	1.445	<0.001	1.288	1.115	1.458	<0.001
Cirrhosis								
Without	Reference				Reference			
With	1.332	1.151	1.451	< 0.001	1.295	1.118	1.462	<0.001
Urbanization level								
1 (the highest)	1.270	1.154	1.352	<0.001	1.252	1.161	1.368	<0.001
2	1.167	1.098	1.221	<0.001	1.174	1.107	1.226	<0.001
3	1.074	0.992	1.122	0.078	1.074	0.981	1.114	0.308
4 (the lowest)	Reference				Reference			
Level of care								
Hospital center	1.226	1.153	1.291	<0.001	1.169	1.119	1.231	<0.001
Regional hospital	1.152	1.083	1.198	<0.001	1.121	1.050	1.194	<0.001
Local hospital	Reference				Reference			

The abbreviations: adjusted hazard ratio (aHR); confidence interval (CI); hepatocellular carcinoma (HCC); hepatitis B (HBV); hepatitis C (HCV); coronary artery disease (CAD); cerebrovascular disease (CVD); New Taiwan dollar (NTD); diabetes mellitus (DM); hypertension (HTN); chronic obstructive pulmonary disease (COPD); chronic kidney disease (CKD).

**Table 3:** Years to CAD.

HCC Patients	Min	Median	Max	Mean ± SD	*p*	Scheffe Post Hoc
(1) non-HBV, non-HCV	0.01	4.89	17.68	5.05 ± 5.15		
(2) HBV only	0.01	5.03	17.34	5.27 ± 5.31		
(3) HCV only	0.01	4.35	17.30	4.44 ± 4.57		
(4) HBV and HCV	0.01	4.67	17.18	4.89 ± 4.92		
Overall	0.01	4.78	17.72	5.05 ± 5.12	0.018	2 > 1 > 4 > 3

*p*: One-way ANOVA with Scheffe post hoc test. The abbreviations: hepatocellular carcinoma (HCC); hepatitis B (HBV); hepatitis C (HCV); coronary artery disease (CAD); standard deviation (SD).

**Table 4:** Years to CVD.

HCC Patients	Min	Median	Max	Mean ± SD	*p*	Scheffe Post Hoc
(1) non-HBV, non-HCV	0.01	4.82	17.65	5.02 ± 5.13		
(2) HBV only	0.01	4.99	17.31	5.23 ± 5.28		
(3) HCV only	0.01	4.27	17.26	4.41 ± 4.50		
(4) HBV and HCV	0.01	4.63	17.12	4.82 ± 4.87		
Overall	0.01	4.74	17.65	5.02 ± 5.09	0.007	2 > 1 > 4 > 3

*p*: One-way ANOVA with Scheffe post hoc test. The abbreviations: hepatocellular carcinoma (HCC); hepatitis B (HBV); hepatitis C (HCV); cerebrovascular disease (CVD); standard deviation (SD).

## Data Availability

All data generated or analyzed during this study are included in this published article.
